# Concurrent *Sphingomonas paucimobilis* and *Mycobacterium tuberculosis* Meningitis in an Immunocompromised Patient: A Rare Case Report and Comprehensive Review of Literature

**DOI:** 10.3390/medicina59040687

**Published:** 2023-03-30

**Authors:** Iosif Marincu, Felix Bratosin, Iulia Bogdan, Catalin Dumitru, Carmen Nicoleta Stoica, Andrei Nicolae Csep, Narcisa Mederle, Roxana Manuela Fericean, Alexandru Ovidiu Mederle, Reshmanth Prathipati, Gratiana Nicoleta Chicin, Adelina Mavrea, Paula Irina Barata, Adrian Vasile Bota

**Affiliations:** 1 Methodological and Infectious Diseases Research Center, Department of Infectious Diseases, “Victor Babes” University of Medicine and Pharmacy, Eftimie Murgu Square 2, 300041 Timisoara, Romania; 2 Doctoral School, “Victor Babes” University of Medicine and Pharmacy, Eftimie Murgu Square 2, 300041 Timisoara, Romania; 3 Department of Obstetrics and Gynecology, “Victor Babes” University of Medicine and Pharmacy Timisoara, Eftimie Murgu Square 2, 300041 Timisoara, Romania; 4 Department of Surgery, Multidisciplinary Center for Research, Evaluation, Diagnosis and Therapies in Oral Medicine, “Victor Babes” University of Medicine and Pharmacy Timisoara, Eftimie Murgu Square 2, 300041 Timisoara, Romania; 5 Santiram Medical College and General Hospital, Nandyala 518001, India; 6 Faculty of General Medicine, “Vasile Goldis” Western University of Arad, Bulevardul Revolutiei 94, 310025 Arad, Romania; 7 National Institute of Public Health, Strada Doctor Leonte Anastasievici 1-3, 050463 Bucuresti, Romania; 8 Department of Internal Medicine I, Cardiology Clinic, “Victor Babes” University of Medicine and Pharmacy Timisoara, Eftimie Murgu Square 2, 300041 Timisoara, Romania; 9 Department of Physiology, Faculty of Medicine, “Vasile Goldis” Western University of Arad, Revolutiei Square 94, 310025 Arad, Romania

**Keywords:** meningitis, *Sphingomonas paucimobilis*, *Mycobacterium tuberculosis*

## Abstract

*Sphingomonas paucimobilis* is a gram-negative bacillus that is widely distributed in the environment but rarely causes infections in humans. Meningitis caused by *S. paucimobilis* is an extremely rare clinical entity with very few reported cases in the literature. The clinical presentation and management of *S. paucimobilis* meningitis are not well established, and further research is needed to better understand this rare infection. Therefore, the goal of this study was to present probably the only case of meningitis caused by co-infection with *S. paucimobilis* and *Mycobacterium tuberculosis* and to describe the diagnostic and therapeutic challenges encountered, in correlation with the other very few reported cases of *S. paucimobilis* meningitis. A 64-year-old male farmer residing in a rural area was admitted with symptoms of severe headache, somnolence, and confusion. He had several comorbidities, including adrenal insufficiency, duodenal ulcer, and hypercholesterolemia. Lumbar puncture showed elevated leukocyte counts, glucose, and a marked rise of cerebrospinal fluid (CSF) proteins, indicating bacterial meningitis, which was confirmed by CSF culture that isolated *S. paucimobilis* and *Mycobacterium tuberculosis*. Antituberculosis therapy was initiated with isoniazid (300 mg/day), rifampicin (600 mg/day), pyrazinamide (2000 mg/day), and streptomycin (1 g/day). Ceftriaxone was introduced nine days later, after CSF culture grew *S. paucimobilis*, and was discharged without complications after 40 days of hospitalization. The literature search revealed a total of 12 published cases of *S. paucimobilis* meningitis in patients ranging from two months old to 66 years old. Among these cases, eight (66%) reported a favorable outcome, while two (17%) cases resulted in a poor outcome, and two (17%) were fatal. It was observed among the 13 identified cases (including ours) that the CSF white blood cell count had an average of 178.9 × 10^3^/mm^3^, an average glucose level of 33.0 mg/dL, and an average protein count of 294.2 mg/dL. Most cases improved appropriately under antibiotic therapy with intravenous ceftriaxone, Meropenem, and Vancomycin. In conclusion, although extremely rare, *S. paucimobilis* meningitis has good outcomes even in immunocompromised patients with appropriate antibiotic therapy and close monitoring, while the diagnosis should not be excluded even in immunocompetent patients.

## 1. Introduction

Bacterial meningitis requires prompt diagnosis and treatment to minimize morbidity and mortality. The most common bacterial causes of meningitis include *Streptococcus pneumoniae*, *Neisseria meningitidis*, and *Haemophilus influenzae*. However, less common bacterial causes can also lead to meningitis, especially in immunocompromised patients. Examples of less common bacterial causes are *Listeria monocytogenes*, *Escherichia coli*, *Staphylococcus aureus*, *Streptococcus agalactiae*, and other gram-negative bacilli [1]. Nevertheless, in rare instances, meningitis can be caused by concurrent infections. *Sphingomonas paucimobilis* is an aerobic gram-negative, non-sporing bacillus that is emerging as an opportunistic pathogen ubiquitous in both the natural environment (water and soil) and hospital settings that rarely infects healthy individuals [2,3,4]. The epidemiology of *Sphingomonas paucimobilis* meningitis is not well-defined, primarily because it is a rare condition. It usually occurs in patients who are immunocompromised, such as those with cancer, chronic lung disease, or organ transplantation, or those undergoing invasive procedures, such as catheterization [5]. Nevertheless, there have been a few reported cases of *Sphingomonas paucimobilis* meningitis in healthy individuals [6,7].

The treatment of *Sphingomonas paucimobilis* meningitis typically involves a combination of antimicrobial therapy and supportive care [8]. The choice of antibiotics depends on the susceptibility of the bacterium, which can be determined by performing susceptibility testing. *Sphingomonas paucimobilis* is generally susceptible to several classes of antibiotics, including beta-lactams, aminoglycosides, and fluoroquinolones [9]. However, it is often resistant to cephalosporins and carbapenems [10]. In some cases, surgical intervention may be necessary to remove infected medical devices, such as shunts or catheters, and the duration of treatment varies depending on the severity of the infection, but it typically lasts for several weeks to months [11]. In immunocompromised patients, the prognosis of *Sphingomonas paucimobilis* meningitis can be poor, with a high mortality rate despite appropriate antimicrobial therapy [12]. Therefore, early recognition and prompt treatment are crucial in improving the outcome of the disease and avoiding complications such as epilepsy, deafness, hydrocephalus, and cognitive deficits [13].

Tuberculosis (TB) is a communicable disease caused by *Mycobacterium tuberculosis* that remains a major public health concern worldwide, particularly in developing countries. However, despite being relatively low in incidence compared to developing countries, TB continues to be a significant issue in developed countries [14]. The epidemiology of TB in developed countries is influenced by various factors, including the availability of diagnostic tools, public health policies, and the level of socioeconomic development [15]. The incidence of TB in developed countries has been declining over the past decades. According to the World Health Organization (WHO), in 2020, the incidence rate of TB in the European Union/European Economic Area (EU/EEA) was 5.5 cases per 100,000 population, which is relatively low compared to developing countries [16]. However, the decline in TB incidence has been slower in developed countries compared to developing countries, and TB elimination targets have not yet been achieved in many developed countries.

TB in developed countries is more commonly reported among specific subpopulations, including migrants, people who inject drugs, and individuals living in crowded settings. Additionally, TB can be reactivated in individuals who had previous exposure to TB, particularly in immunocompromised individuals such as those with HIV infection [17]. Although TB primarily affects the lungs (pulmonary TB), it can also involve other organs and tissues (extrapulmonary TB). Extrapulmonary TB can occur in various locations, including lymph nodes, pleura, genitourinary tract, bones and joints, and the central nervous system. TB can also manifest in atypical locations, such as the oral cavity, where it may present as tuberculous ulcers or granulomatous lesions [18]. The presence of TB in such locations can pose diagnostic challenges, and a high index of suspicion is necessary to identify and initiate appropriate treatment. TB control in developed countries is based on a comprehensive approach that includes active case finding, contact tracing, treatment, and infection control measures. The implementation of these measures varies across countries and is influenced by their public health policies and resource allocation [19].

In HIV-positive patients and other categories of immunocompromised patients, the risk of opportunistic infections increases as the immune system becomes progressively weakened. It is essential to recognize the most common co-existing infections in this population to provide appropriate and timely management. The most common viral infections commonly seen in HIV-positive patients include cytomegalovirus (CMV) infection, herpes simplex virus (HSV) infection, varicella-zoster virus (VZV) infection, human papillomavirus (HPV) infection, and hepatitis B and C virus infections. The main bacterial infections include *Mycobacterium tuberculosis*, *Pneumocystis jirovecii* pneumonia, toxoplasmosis, bacterial pneumonia, and salmonellosis. Lastly, to most common fungal infections include Candidiasis, Cryptococcosis, Histoplasmosis, Coccidioidomycosis, and Aspergillosis [20]. Understanding the interplay between immunity and these opportunistic infections is crucial in managing immunocompromised patients.

*Mycobacterium tuberculosis* meningitis (MTBM) is a rare and severe form of tuberculosis that affects only 1% of all tuberculosis cases, but it causes significant morbidity and mortality, particularly in developing countries [21]. The epidemiology of MTBM is influenced by various factors, including HIV co-infection, age, and immune status. MTBM predominantly affects young children and immunocompromised individuals, such as those living with HIV or undergoing immunosuppressive therapy [22]. HIV co-infection is a significant risk factor for developing MTBM, with up to 50% of HIV-positive patients with TB developing MTBM [23]. Additionally, the incidence of MTBM is higher in older adults, particularly those aged over 50 years [24]. Despite the availability of effective treatments, the mortality rate of MTBM remains high, particularly in developing countries, where access to diagnostic tools and treatments may be limited. Therefore, early diagnosis, prompt treatment, and appropriate supportive care are crucial in improving the outcomes of MTBM.

The hypothesis of the current study is that meningitis caused by co-infection with *Sphingomonas paucimobilis* and *Mycobacterium tuberculosis* in an immunocompromised patient is a rare occurrence that poses significant diagnostic and therapeutic challenges. Therefore, the goals of this study were to present a rare case of meningitis caused by co-infection with *Sphingomonas paucimobilis* and *Mycobacterium tuberculosis* in an immunocompromised patient and to describe the diagnostic and therapeutic challenges encountered. A secondary objective is to provide a comprehensive review of the existing literature on the cases of meningitis with these two pathogens or single infection with *Sphingomonas paucimobilis*, including epidemiology, pathogenesis, clinical presentation, diagnosis, and management while discussing the possible mechanisms of co-infection and the potential implications for clinical practice and future research.

## 2. Case Presentation

### Case Report

We present a case report of a 64-year-old male farmer residing in a rural area who was admitted to the Neurology Clinic of Timisoara County Hospital. The patient exhibited symptoms of severe headache, somnolence, and confusion. The patient was transferred to the Clinic of Infectious Diseases the following day with a suspicion of acute meningoencephalitis. The patient’s medical history revealed several comorbidities, including adrenal insufficiency of unknown etiology, for which oral hydrocortisone was prescribed, duodenal ulcer with *Helicobacter pylori* infection being treated with 40 mg/day esomeprazole, and hypercholesterolemia for which 10 mg rosuvastatin was taken in the evening. The patient presented with severe and persistent headache, incoherent speech, drowsiness, confusion, fever (37.9 °C), and a stiff neck. Two healing skin lesions were noted on the dorsal right hand, attributed to agricultural activities in contact with soil and dirty water. A CT scan of the brain was performed to exclude intracranial tumors, which showed no evidence of increased intracranial pressure. However, magnetic resonance imaging of the brain revealed fluid in the cerebral spaces with no pathological features observed in the supra- and infratentorial parenchyma or median line structures following the administration of the contrast agent.

Lumbar puncture, as described in Table 1, showed elevated leukocyte counts (340 leukocytes/mm^3^), 75% lymphocytes, 25% polymorphonuclear leukocytes, glucose (16 mg/dL), and a marked rise of CSF proteins (386.1 mg/dL), indicating bacterial meningitis, including tuberculous meningitis, which was confirmed by CSF culture. Laboratory tests presented in Table 2 revealed a slightly elevated leukocyte count (13.140/µL) and elevated C-reactive protein levels (CRP) (23.01 mg/L), suggesting a bacterial infection, while the erythrocyte count, hemoglobin, hematocrit, platelets, erythrocyte sedimentation rate, fibrinogen, alanine aminotransferase, aspartate aminotransferase, gamma-glutamyltransferase, alkaline phosphatase, serum urea, serum creatinine, serum uric acid, serum glucose, serum protein electrophoresis, and urinalysis were normal. The patient tested negative for HIV, but Candida albicans was identified from a tongue swab as a side effect of the immunosuppressive effect of cortisone therapy that allows the overgrowth of *Candida* spp. that is normally a commensal oral fungus. Chest radiographs were conducted to detect pulmonary changes useful for diagnosing tuberculosis, but the findings were normal.

To isolate *Sphingomonas paucimobilis*, a cerebrospinal fluid (CSF) culture was performed using blood and chocolate agar medium, while the antibiotic sensitivity was conducted through an automated Vitek-2 Compact method. The identification of *Mycobacterium tuberculosis* was based on the macroscopic appearance of colonies obtained from CSF culture on the Löwenstein–Jensen medium and positive results from the Ziehl–Neelsen immunochromatographic test. Identification of Candida species was also performed by using the Vitek-2 system and the Sabouraud culture growth. Standard biochemical and bacteriological protocols for CSF processing were followed using the Cobas Integra 400 Plus analyzer in the laboratory. Even though the patient had a history of duodenal ulcer with *H. pylori* infection, there were no attempts to isolate *H. pylori* in the collected samples.

Based on the clinical presentation of fever (37.9 °C), moderate leukocyte levels in cerebrospinal fluid (CSF), the predominance of lymphocytes, marked increase in CSF protein, and accentuated decrease of CSF glucose, antituberculosis therapy was initiated with isoniazid (300 mg/day), rifampicin (600 mg/day), pyrazinamide (2000 mg/day), and streptomycin (1 g/day). To supplement specific therapy, 10% glucose, 20% mannitol, and paracetamol were administered. On the third day of hospitalization, oral hydrocortisone therapy was substituted with intravenous (I.V.) hydrocortisone (3 × 100 mg/day) after an endocrinological consultation. This substitution was made to improve the properties of the blood–brain barrier, as described in the existing literature [25]. Despite initial therapy, the patient experienced irregular fever, fatigue, headaches, and drowsiness. On the ninth day of hospitalization, CSF culture showed a growth of *Sphingomonas paucimobilis* susceptible to ceftriaxone, ceftazidime, Cefepime, ciprofloxacin, pefloxacin, amikacin, gentamicin, tobramycin, imipenem, Meropenem, trimethoprim-sulfamethoxazole, ticarcillin, piperacillin, and ampicillin/sulbactam. Antituberculosis therapy was stopped on the 10th day and replaced with ciprofloxacin (2 × 6 mg/day, I.V.), Cefepime (4 g/day, I.V.), 20% mannitol, 10% glucose, hydrocortisone, ranitidine, paracetamol, and fluconazole for candida infection.

The patient continued to exhibit symptoms such as irregular fever, fatigue, intermittent headaches, and night sweats. Control lumbar puncture on day 16 revealed a decrease in leukocyte counts, a slight reduction in polymorphonuclear leukocyte count, a decrease in CSF protein, and a decrease in lymphocyte counts, indicating improvement of the meningoencephalitis. On day 24, the control lumbar puncture showed a decrease in CSF leukocytes and a polymorphonuclear leukocyte count increase in lymphocytes but low CSF glucose. The patient was monitored and tested positive for urinary infection with tropicalis, susceptible to amphotericin B, nystatin, ketoconazole, fluconazole, and flucytosine. Fluconazole (200 mg/day) was added to the treatment regimen. The patient continued to exhibit symptoms, with a cough and low fever, and a pulmonary examination revealed dullness on the right hemithorax. The chest radiograph showed a small quantity of pleural effusion in the right basal lung. A pleural puncture was performed, and antituberculosis therapy was reintroduced after a culture of the pleural fluid revealed *Mycobacterium tuberculosis*. Antibiotic treatment was interrupted on the 34th day after the CSF culture was sterile, but antituberculosis therapy was continued. After six more days of clinical and therapeutic surveillance, the patient was afebrile, in good general condition, and requested to be discharged to continue antituberculosis therapy at home under the care of physicians from the territorial center for control and surveillance of TB.

## 3. Discussion

The results of the literature search conducted revealed a total of 12 published cases of meningitis attributed to *Sphingomonas paucimobilis*. Among these cases, eight (66%) reported a favorable outcome, while two (17%) cases resulted in a poor outcome, and two (17%) were fatal. Given this information, it is noteworthy that our current case represents the first reported instance of co-infection with both *S. paucimobilis* and *Mycobacterium tuberculosis* in the medical literature. Moreover, it is important to highlight that our case stands out from the two previous cases in the literature, as it resulted in a favorable outcome. This outcome is particularly noteworthy considering the double bacterial etiology. Consequently, we consider this case to be a therapeutic success, as it highlights the effectiveness of the treatment administered in combating this rare and challenging condition.

*Mycobacterium tuberculosis*, the causative agent of tuberculosis, is transmitted primarily through respiratory droplets from an infected person. It is a common infection in areas with high population density, poor sanitation, and inadequate healthcare facilities [26]. This patient did not have a history of working in a healthcare setting or living in close contact with someone who has tuberculosis, which could put the patient at risk of contracting the infection. On the other side, *Sphingomonas paucimobilis* is an environmental bacterium commonly found in soil and water [27]. Considering the patient was living in a rural region, it is possible that the patient was exposed to the bacterium through contact with contaminated soil or water or through an invasive medical procedure. A more comprehensive understanding of the patient’s environment and medical history would be needed to determine the exact sources of these infections.

### 3.1. First Case—Hajiroussou V. et al. [6]

In 1979, Hajiroussou et al. reported the initial instance of meningitis caused by *S. paucimobilis* [5]. The patient, a 33-year-old male farmer who was previously engaged in soil-based labor, presented with a wound susceptible to infection on his leg and a history of epilepsy treated with phenobarbitone and phenytoin. The patient presented with symptoms of meningitis, including headache, neck stiffness, and fever. His cerebrospinal fluid (CSF) analysis revealed increased white blood cell count with 95% lymphocytes, normal glucose levels, and elevated protein levels. A CSF culture was positive for *Pseudomonas paucimobilis*, that was later renamed as *Sphingomonas paucimobilis*. The patient was treated with intramuscular streptomycin 1 g and showed improvement in his symptoms, being discharged after ten days of hospitalization. Since the first published case, there has been a scarcity of data regarding the patients diagnosed with meningitis with *Sphingomonas paucimobilis*, with more cases being published after 2010, as described in Table 3.

### 3.2. Second Case—Tai ML et al. [28]

A 31-year-old previously healthy man with no previous medical illness presented to the emergency department at a tertiary hospital in Malaysia with fever and headache for three weeks in May 2012. The patient had a history of loss of appetite and weight loss for two months and became more lethargic and spoke irrelevantly. One day prior to admission, the patient had a change in his behavior. He had seen the general practitioners twice over the course of three weeks, but the administration of antibiotics was unknown. A lumbar puncture was performed, which showed a high opening pressure and increased white blood cells, glucose, and protein levels in the cerebrospinal fluid, consistent with meningitis. The patient was given intravenous ceftriaxone and acyclovir, and anti-tuberculous medication was given to cover tuberculous meningitis. Chest X-ray was normal, and a plain brain CT scan conducted after the lumbar puncture showed an unchanged degree of hydrocephalus and cerebral edema. The patient’s GCS score fluctuated from 9/15 to 7/15, and he was intubated and ventilated. Despite being planned for extraventricular drainage (EVD) by the neurosurgical team, the patient passed away the following day. The case highlights the importance of early recognition and treatment of *S. paucimobilis* meningitis, which can progress rapidly and lead to severe complications such as cerebral edema and hydrocephalus. It also emphasizes the importance of appropriate antibiotic therapy, especially in immunocompromised patients.

### 3.3. Third Case—Bolen RD et al. [29]

A 39-year-old woman with a history of end-stage renal disease and a deceased donor kidney transplant presented to the nephrology transplant clinic with severe headache, dizziness, nausea, neck pain, gait imbalance, and recent hearing loss. The patient was on immunosuppressive therapy with mycophenolic acid, tacrolimus, and prednisone. Neurological examination revealed deficits of short-term memory, bilateral horizontal gaze nystagmus, hyperreflexia, bilateral Babinski sign, and meningismus. Laboratory evaluation showed mild leukopenia and hyponatremia. Lumbar puncture revealed high opening pressure and cerebrospinal fluid analysis consistent with ventriculitis. Magnetic resonance imaging of the brain showed diffuse periventricular T2 hyperintensities along the lateral ventricles and the third ventricle. The patient was treated with empiric antibiotic therapy and dexamethasone. Ganciclovir was added to the regimen for empiric treatment of possible CMV ventriculitis, given the immunocompromised state. The patient eventually required intubation and mechanical ventilation. Cerebrospinal fluid cultures grew *S. paucimobilis*, and the antimicrobial regimen was tailored accordingly. The organism was identified using matrix-assisted laser desorption/ionization-time of flight mass spectrometry, and antimicrobial susceptibility testing was performed using the automated Microscan System. The patient was eventually treated with Meropenem for a total of three weeks and eventually made a full recovery. The case highlights the importance of appropriate antibiotic therapy and the use of continuous video EEG monitoring in patients with severe central nervous system infections. The case suggests that *S. paucimobilis* should be considered in the differential diagnosis of central nervous system infections in immunocompromised patients.

### 3.4. Fourth Case—Deveci N. et al. [30]

This report describes the case of a 14-year-old male student who presented to a hospital with a fever, headache, and vomiting. The patient had no underlying health conditions, complete vaccinations, and no prior hospitalization. Physical examination showed hyperactive deep tendon reflexes, positive meningeal irritation findings, and a preliminary diagnosis of meningitis was established. Lumbar puncture revealed turbid cerebrospinal fluid (CSF) with increased pressure, a high cell count, high protein levels, and low glucose levels. Further tests were conducted to rule out other infections, including real-time PCR for enterovirus, adenovirus, herpes simplex virus type 1, and *Mycobacterium tuberculosis* complex, which were found to be negative. The HIV test was negative, as well as the blood culture. The cranial MR imaging revealed meningeal contrasting in the frontal region, T2 signal increase, and parasinusitis due to mucosal thickening and leveling secondary to infection. The patient was treated with dexamethasone, Vancomycin, and ceftriaxone. The aerobic bacterial culture of the CSF grew *Sphingomonas paucimobilis*, which was sensitive to various antibiotics. The patient recovered after 48 h, and antibiotic treatment was completed in 14 days, after which the patient was discharged and referred for follow-up.

### 3.5. Fifth Case—Göker T. et al. [13]

A previously healthy 48-year-old woman presented to the emergency department with sudden-onset speech disturbance and worsening general status. Neurological examination revealed unconsciousness, abnormal eye movement, and an abnormal response to painful stimuli. A computerized brain tomography showed a hemorrhagic lesion in the basal ganglia leading to hydrocephalus. An external ventricular drain was placed, and daily CSF smears and cultures were sent. On the 23rd day, gram-negative bacilli were seen in CSF gram staining, and *S. paucimobilis* was identified in CSF culture. Antibiotic susceptibility testing showed resistance to colistin but sensitivity to Meropenem and gentamycin. The patient was treated with Meropenem, and no evidence of infection was detected on follow-up brain CTs. Prophylactic ceftriaxone therapy was also administered during the same period, and wound and catheter care was repeated with a new external ventricular drainage catheter placed every ten days. No permanent ventriculoperitoneal shunt was placed since the patient could not tolerate closure of the drainage due to hydrocephalus. Tests for immune insufficiency were negative, and immunoglobulin levels were within normal range. However, the patient died from heart failure on the 46th day of admission.

### 3.6. Sixth Case—Mehmood H. et al. [31]

A 50-year-old woman with a history of rheumatoid arthritis presented with headache, dizziness, chills, shakiness, and neck pain with nuchal rigidity that worsened over the course of three to four days. On examination, severe neck rigidity with decreased range of motion was noticed. A computed tomography scan of the neck and a magnetic resonance imaging of the brain was normal. A lumbar puncture was performed, which revealed clear cerebrospinal fluid (CSF) with an opening pressure of 10 mm/Hg, white blood cell count of 5/mm^3^, red blood cell count of 95/mm^3^, protein of 37 mg/dL, and glucose of 60 mg/dL. Gram stain of CSF showed gram-negative rods. Intravenous Meropenem was initiated, and CSF culture grew *Sphingomonas paucimobilis*, which was sensitive to Meropenem. The patient’s symptoms improved after five days of treatment, and she was subsequently discharged with a home health nurse to complete a 21-day course of intravenous Meropenem. At the follow-up five weeks later, the patient had completely recovered with no neck pain or rigidity.

### 3.7. Seventh Case—Ciftci N. et al. [32]

A 13-year-old girl with a history of ventriculoperitoneal shunt following posterior fossa tumor surgery ten years prior presented to the emergency room with fever and headache for two days. She was diagnosed with meningitis, and a cerebrospinal fluid (CSF) sample was taken before empirical treatment with Vancomycin and Meropenem. The shunt was removed, and CSF was drained externally. After incubation of triplicated CSF samples, slow-growing yellow-pigmented gram-negative bacilli were isolated and identified as *S. paucimobilis* using the VITEK 2 automated system. Antimicrobial susceptibility testing showed susceptibility to imipenem, colistin, levofloxacin, Meropenem, and Cefepime. Despite treatment, CSF culture continued to reproduce with *S. paucimobilis* after three weeks, leading to the addition of levofloxacin to the treatment regimen and shunt revision. The patient was successfully treated with appropriate antibiotics.

### 3.8. Eighth Case—Orozco-Hernández J. et al. [33]

A three-year-old boy presented to the hospital with a high fever, headache, vomiting, and neck stiffness. He was previously healthy with no history of recent travel or exposure to sick contacts. Upon physical examination, the patient was found to have nuchal rigidity, positive Kernig’s and Brudzinski’s signs, and a mild paresis of the right upper limb. Cerebrospinal fluid (CSF) analysis revealed an elevated white blood cell count, low glucose levels, and a high protein level, indicating bacterial meningitis. Gram stain of the CSF showed gram-negative rods, and further microbiological testing identified the pathogen as *S. paucimobilis*. The patient was immediately started on intravenous antibiotics, including ceftriaxone and Vancomycin. He also received supportive care, including antipyretics, hydration, and pain management. His fever subsided within the first 48 h of treatment, and his neurologic symptoms gradually improved. Repeat CSF analysis after ten days of treatment showed a normal cell count and glucose level and no growth of bacteria on culture. The patient was discharged from the hospital after 14 days of treatment with a good overall outcome. No neurologic deficits were noted during follow-up visits, and the patient’s development continued to progress normally.

### 3.9. Ninth Case—Muhyi A. et al. [34]

A two-month-old infant was admitted to the emergency department with convulsive status epilepticus with fever. On neurological examination, the patient had generalized seizures, an irritable mood, and upper motor neuron lesions. Laboratory tests revealed leukocytosis, an elevated platelet level, and an unclear cerebrospinal fluid with high cell count, mononuclear and polymorphonuclear cells, protein, and low glucose levels. A computerized brain tomography revealed an empyema subdural. The cerebrospinal fluid culture showed *S. paucimobilis* sensitive to several antibiotics. The patient was treated with Meropenem for 14 days and underwent burr-hole evacuation surgery by the neurosurgeon. The patient showed significant improvement and was discharged to home. Outpatient care showed no complaints of seizure, fever, or neurological disorders. Rapid diagnosis and early surgical intervention are critical for improving patient outcomes. The patient’s response to Meropenem supports the use of this antibiotic in treating *S. paucimobilis* infections in pediatric patients.

### 3.10. Tenth Case—Ohnmar O. et al. [35]

A 44-year-old man with end-stage renal disease due to diabetic nephropathy on regular hemodialysis and chronic hepatitis B infection presented with fever, loose motion, and confusion for two days. On admission to a private hospital, he was unconscious, hypotensive, and had a Glasgow Coma Scale (GCS) of four. Laboratory tests revealed leukocytosis, elevated creatinine and urea, and positive HBs antigen, anti-HBe, and anti-HBc. Imaging studies were unremarkable, and initial cultures were negative. He was diagnosed with septic and uremic encephalopathy and treated with intravenous Vancomycin and more intensive hemodialysis. His GCS mildly improved, and he was transferred to another hospital where further workup revealed positive CSF culture for *S. paucimobilis* and multiple hyperintensities in the bilateral periventricular/subcortical regions on MRI, suggestive of acute disseminated encephalomyelitis (ADEM). His antibiotics were changed for Cefepime according to sensitivity, and intravenous methylprednisolone was initiated for ADEM. His GCS significantly improved after five days of steroid initiation. He was discharged with a GCS of 15 but remained chair-bound.

### 3.11. Eleventh Case—Fernández-Sarrateaa MP et al. [36]

A 30-year-old male presented with headache and vertigo for seven days and was treated for bacterial pharyngitis without improvement. He later developed behavioral disturbances, neck pain, and neck stiffness. Blood tests showed high white cell count, neutrophils, and erythrocyte sedimentation rate. Empiric antibiotic therapy was prescribed for suspected meningoencephalitis with ceftriaxone, Vancomycin, and acyclovir. A lumbar puncture revealed cloudy cerebrospinal fluid with high white blood cell count, neutrophils, low glucose, and high protein. Gram-staining showed gram-negative rods, and the CSF culture identified *S. paucimobilis* with resistance to ceftriaxone, Cefepime, and aztreonam and susceptibility to other antibiotics. On day eight of hospitalization, the patient developed intracranial hypertension, which was found to be caused by hydrocephalus requiring a ventriculoperitoneal shunt. After two weeks of antibiotic treatment, the patient was discharged from the hospital after a 14-day-long course of Meropenem, and his mental function returned to normal. After five weeks of follow-up, the patient was asymptomatic and reinstated to his normal activities.

### 3.12. Twelveth Case—Bae SW, Lee JH [37]

A 66-year-old woman presented with a fever, neck stiffness, and slight drowsiness for five days. She had a history of receiving more than 100 acupuncture treatments and moxibustion cupping on both sides of her posterior neck eight days before admission. She had no gastrointestinal symptoms but had a history of breast cancer and received her final adjuvant chemotherapy thirteen months prior to admission. On admission, the patient had high blood pressure, an elevated white blood cell count in cerebrospinal fluid, and a turbid appearance of the fluid. The initial cerebrospinal fluid analysis revealed bacterial meningitis caused by *S. paucimobilis* and *L. monocytogenes*, and the patient was treated with antibiotics and dexamethasone sodium phosphate. However, her condition deteriorated, and she underwent an emergency external ventricular drainage for hydrocephalus. Later, she had a sudden deterioration of her mental status and received a second external ventricular drainage insertion and a ventriculoperitoneal shunt. After 91 days of hospitalization, the patient was transferred to a rehabilitation hospital with improved motor function and cognitive status.

### 3.13. Summary of Findings

**Figure 1:**
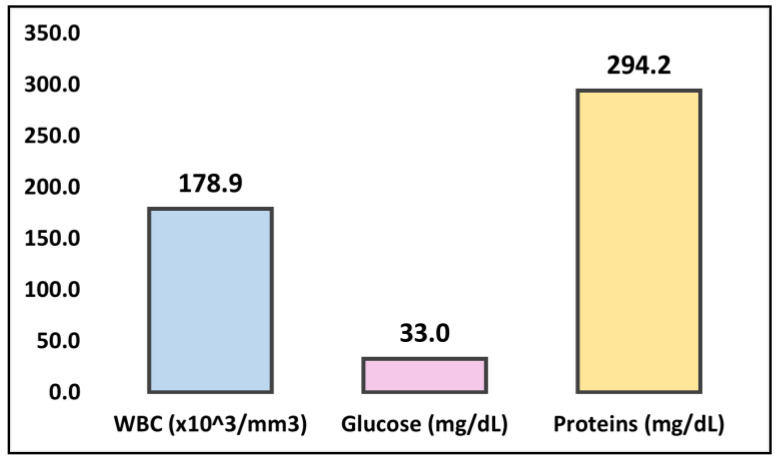
Summary of findings in CSF samples.

The following cases describe patients who were diagnosed with meningitis caused by *Sphingomonas paucimobilis*, a gram-negative bacterium commonly found in the environment but rarely associated with human infections. The cases highlight the importance of early recognition and treatment of this pathogen, which can progress rapidly and lead to severe complications such as cerebral edema and hydrocephalus. The clinical presentation of *Sphingomonas paucimobilis* meningitis varied among the cases, with common signs and symptoms including fever, headache, neck stiffness, nausea, vomiting, and altered mental status. Laboratory findings showed elevated white blood cell counts, low glucose levels, and high protein levels in the cerebrospinal fluid, consistent with bacterial meningitis. It was observed among the 13 identified cases (including ours) that the white blood cell count had an average of 178.9 mm^3^/mm^3^, an average glucose level of 33.0 mg/dL, and an average protein count of 294.2 mg/dL, as presented in Figure 1.

Although many patients were immunocompetent, more than half of all patients had predisposing comorbidities and risk factors such as immunosuppression, end-stage renal disease, or HIV. The diagnosis was established through the lumbar puncture and subsequent culturing of the cerebrospinal fluid, which grew *Sphingomonas paucimobilis*. In some cases, other diagnostic tests such as brain imaging, real-time PCR for other infectious agents, and video EEG monitoring were also performed to rule out other potential causes or complications. Treatment involved the administration of appropriate antibiotic therapy, including empiric intravenous ceftriaxone, Meropenem, Vancomycin, or other antimicrobials once the susceptibility testing results are available. In some cases, antiviral medication was also given to cover possible viral causes of meningitis. Some patients required intubation and mechanical ventilation, as well as surgical interventions such as external ventricular drainage or ventriculoperitoneal shunt placement for hydrocephalus. The duration of hospitalization varied among the cases, with some patients recovering within a few days to several weeks, while others required longer hospital stays due to complications or comorbidities. The survival rate was generally favorable, with most patients making a full recovery without any neurological deficits.

Considering the identified cases, clinicians should consider several aspects if they encounter a similar situation in their hospital practice. Recognition of potential co-infections is a main point. In this case, the patient was co-infected with both *Sphingomonas paucimobilis* and *Mycobacterium tuberculosis*, which is the first reported instance in the medical literature. Clinicians should be vigilant in considering co-infections, especially in patients with complex medical histories and multiple risk factors. Also, the utilization of appropriate diagnostic tests, such as lumbar puncture, imaging, and CSF culture, played a crucial role in identifying the causative organisms in this case. Timely identification of the co-infections allowed for appropriate treatment and resulted in a favorable outcome. Also, the identification of *Candida albicans* from a tongue swab in a patient undergoing cortisone therapy is not uncommon, and it is important for clinicians to monitor for the development of Candida-related infections in these patients. Lastly, adapting and modifying treatment based on clinical progress and laboratory findings is a key case management decision.

## 4. Conclusions

An immunocompromised farmer was diagnosed with acute meningoencephalitis caused by co-infection with *S. paucimobilis* and *M. tuberculosis*, which is unusual in the medical literature, as it is probably the first reported case of this kind. In this case, the treatment was modified multiple times based on the patient’s clinical progress and laboratory findings, including the switch from antituberculosis therapy to antibiotic therapy targeting *Sphingomonas paucimobilis* and later reintroducing antituberculosis therapy. This highlights the importance of closely monitoring the patient’s response to treatment and making necessary adjustments to optimize outcomes. Nevertheless, if a patient with a history of TB presents with signs and symptoms of meningitis, the initiation of antituberculosis therapy should be considered for initiation along with the empirical treatment for meningitis. While *Sphingomonas* spp. infections are uncommon, there has been an increase in reported patients in recent years, where the most severe complications identified were cerebral edema and ventriculomegaly, although with a low mortality rate and good outcomes after antibiotic and supportive therapy. The presented cases suggest that *S. paucimobilis* should be considered even in immunocompetent individuals. The management is based on appropriate broad-spectrum empirical antibiotic therapy for bacterial meningitis until the etiology of infection is established and antibiotic sensitivity testing has been completed, as there are no definitive guidelines for antibiotic treatment for *S. paucimobilis*. Although Ceftriaxone and Meropenem were the most commonly used antibiotics in the presented cases confirmed with *S. paucimobilis* meningitis that survived, more data is necessary to support this treatment scheme.

## Figures and Tables

**Table 1:** Cerebrospinal fluid analysis.

Lab Test	Normal Range	Day 2	Day 16	Day 24	Day 31
WBC count (×10^3^/µL)	<4	340	240	104	100
PMN (%)	0–5%	25	35	20	15
Lymphocytes (%)	20–40%	75%	65%	80%	85%
Erythrocyte (mm^3^)	<5 cells	1300	2400	1700	1200
Glucose (mg/dL)	40–70	16	17	18	28
Protein (mg/dL)	15–45	386.1	305.3	94.9	94.2
Gram stain	-	Neg.	Neg.	Neg.	Neg.
Culture	-	*S.p*+	-	-	*M.t*+

WBC—White blood cells; PMN—Polymorphonuclear cell count; *S.p.*—*Sphingomonas paucimobilis*; *M.t.*—*Mycobacterium tuberculosis*; “+”—positive result; “-”—negative result.

**Table 2:** Laboratory investigations.

Lab Test	Normal Range	Day 2	Day 9	Day 16	Day 24	Day 29	Day 31	Day 33
WBC count (×10^3^/µL)	4.0–10.0	13.14	14.29	13.77	13.52	13.04	11.83	11.26
Erythrocytes (×10^6^/µL)	4.7–5.6	3.7	3.7	3.6	3.6	3.6	3.7	3.7
CRP (mg/L)	0–5	23.01	15.13	14.80	14.51	12.39	12.75	12.10
ESR (mm/1 h)	3–10	18.3	22.6	23.2	21.6	20.1	18.7	18.3
Hb (g/dL)	14–17.2	10.2	10.4	10.5	10.5	10.6	10.6	10.7
Ht (%)	42–51	31	35	34	35	39	39	39

CRP—C-reactive protein; WBC—White blood cells; ESR—Erythrocyte sedimentation rate; Hb—Hemoglobin; Ht—Hematocrit.

**Table 3:** Case reports of *Sphingomonas paucimobilis* meningitis.

Author	Year	Immune Status	Clinical Presentation	Age and Comorbidities	Antibiotic Treatment (Dose and Duration)	Laboratory Findings	Outcome
Hajiroussou V. et al. [6]	1979	Unknown	Fever, headache, stiff neck, vomiting	39 yo male with a history of epilepsy	Streptomycin 1 g/day I.M.	GNB identified in CSF; increased WBC (200 × 10^3^/mm^3^)—75% lymphocytes, glucose (67 mg/dL), and elevated protein levels (40 mg/dL)	Survived, discharged after 10 days
Tai ML et al. [28]	2014	Immunocompetent	Headache, fever, vomiting, behavioral changes, cerebral edema	31 yo male with a history of neurosurgery	Ceftriaxone (2 g/day) I.V. and Acyclovir	GNB identified in CSF; increased WBC (210 × 10^3^/mm^3^)—12% lymphocytes, glucose (31 mg/dL), and elevated protein levels (28 mg/dL)	Died after 2 days of hospitalization
Bolen RD et al. [29]	2015	Immunocompromised	Headache, vomiting, photophobia	39 yo female with a kidney transplant	Vancomycin, Ceftriaxone, Ampicillin, switched to Meropenem (2 g/day) for 3 weeks	*S. paucimobilis* identified in CSF; WBC (78 × 10^3^/mm^3^)—36% lymphocytes, glucose (18 mg/dL), and elevated protein levels (279 mg/dL)	Survived, discharged after 21 days
Deveci N. et al. [30]	2017	Pediatric immunocompetent	Headache, neck stiffness, fever	14 yo male	Ceftriaxone (100 mg/kg/day, Vancomycin (60 mg/kg/day)	GNB identified in CSF; WBC (108 × 10^3^/mm^3^)—10% lymphocytes, glucose (<10 mg/dL), and elevated protein levels (401/dL)	Survived, discharged after 14 days
Göker T. et al. [13]	2017	Immunocompetent	Sudden speech difficulties and a worsening general condition; no signs of meningitis	48 yo healthy female	Ceftriaxone, Meropenem (1000 mg, 3/day)	GNB identified in CSF	Died after 46 days of hospitalization
Mehmood H. et al. [31]	2018	Immunocompetent	Headache, nuchal rigidity, dizziness, no fever	50 yo female	Meropenem for 21 days	*S. paucimobilis* identified in blood and CSF; WBC (50 × 10^3^/mm^3^), glucose (60 mg/dL), and elevated protein levels (37 mg/dL)	Survived and discharged after 5 days
Ciftci N. et al. [32]	2018	Pediatric immunocompetent	Fever, headache, vomiting	13 yo female with a VP shunt	Empiric Vancomycin and Meropenem	*S. paucimobilis* identified in CSF	Survived
Orozco-Hernández J. et al. [33]	2019	Pediatric immunocompetent	Fever, nuchal rigidity, positive Kernig’s and Brudzinski’s sign	3 yo male	Ceftriaxone and Vancomycin	*S. paucimobilis* identified in CSF	Survived and discharged after 14 days
Muhyi A. et al. [34]	2021	Unknown	Epileptic seizures and fever	2-month-old male with a history of head trauma	Meropenem for 14 days	*S. paucimobilis* identified in subdural empyema; WBC (350 × 10^3^/mm^3^), glucose (35 mg/dL), and elevated protein levels (370 mg/dL)	Survived and discharged after 14 days
Ohnmar O. et al. [35]	2021	Immunocompetent	Fever, confusion, coma	44 yo male on hemodialysis	Vancomycin (1 g/day) switched to Cefepime for 3 weeks	*S. paucimobilis* identified in CSF; WBC (20 × 10^3^/mm^3^), glucose (54 mg/dL), and elevated protein levels (14 mg/dL)	Survived and discharged after 1.5 months
Fernández-Sarrateaa MP et al. [36]	2022	Immunocompetent	Headache and dizziness	30 yo male without comorbidities	Ceftriaxone (4 g/day), Vancomycin (30 mg/kg/day), Acyclovir (10 mg/kg/day)—switched to Meropenem	*S. paucimobilis* identified in CSF; WBC (165 × 10^3^/mm^3^), glucose (11 mg/dL), and elevated protein levels (1110 mg/dL)	Survived and discharged after 14 days
Bae SW, Lee JH [37]	2022	Immunocompetent	Fever, neck stiffness, confusion	66 yo female history of breast cancer	Ceftriaxone (2 g/day), Vancomycin (1 g/day) for 14 days, switched to ampicillin/sulbactam and ceftriaxone	*S. paucimobilis* and *Listeria monocytogenes* were identified in CSF and blood, respectively; WBC (429 × 10^3^/mm^3^)—32% lymphocytes, glucose (11 mg/dL), and elevated protein levels (369 mg/dL)	Survived with complications—required VP

NR—Not reported; CSF—Cerebrospinal fluid; yo—years old; WBC—White blood cell count; I.V.—Intravenous; I.M.—Intramuscular; GNB—Gram-negative bacilli.

## Data Availability

Data available on request.
